# Occlusion time analysis in military pilots affected by bruxism

**DOI:** 10.1038/s41598-018-38166-2

**Published:** 2019-02-05

**Authors:** Alessandro Nota, Simona Tecco, Clementina Cioffi, Alessandro Beraldi, Johnny Padulo, Alberto Baldini

**Affiliations:** 1 IRCCS San Raffaele Scientific Institute, Dental School, Vita-Salute San Raffaele University, Milan, Italy; 2 Faculty of Kinesiology, University of Split, Split, Croatia; 3 Institute of Improvement and Training in Aerospace Medicine, Italian Air Force, Rome, Italy; 4 University eCampus, Novedrate, Italy

## Abstract

Military pilots are characterized by peculiar job conditions related to intense accelerative stresses. For this, they frequently report work-related neck and back pain and are affected by bruxism. The aim of this case-control study is to analyze the occlusion time (OT) in a sample of military pilots affected by bruxism, compared with healthy non-pilot subjects. 14 military pilots, all males (mean age 35.14 ± 2.32 y), affected by bruxism, were compared with an age matched control group of 14 male subjects (mean age 32.29 ± 4.84 y). The T-Scan computerized occlusal analysis system (Tek-Scan Inc., Boston, MA, USA) was used to record the subjects’ occlusion times during 4 mandibular opening-closing movements. Military pilots affected by bruxism showed a statistically significant lower (reduced of 85%) mean OT, compared with control subjects (p < 0.0001). Military pilots are characterized by a highly reduced occlusion time compared to healthy non-pilot subjects. This feature could not be only related with the presence of bruxism but with their particular clinical job-related condition that causes different dental and stomatognathic system problems. Further studies are encouraged to enhance the scientific knowledge in this field.

## Introduction

Military pilots are characterized by peculiar pathological conditions related with their daily exposition to intense accelerative stresses^1,2^. For example, these accelerations and the stress of the military flight cause pilots of high-performance fighters frequently report work-related neck and back pain^3–5^ and also different dental and stomatognathic system problems^6^.

For example “Dental Barotrauma” frequently reported during the WWII period, that deals with fracturing of restorations during high-altitude flying^6^ or barodontalgia, which affects 11% of military aircrews at a rate of 5 episodes/1000 flights^7^. Furthermore, probably because of the physical and psychological stresses related with their employment, military pilots are frequently affected by bruxism, disorder characterized by grinding and clenching of the teeth occurring without a functional purpose^8–10^. This was clearly showed in a study published by Lurie *et al*.^11^ reporting that 69% of Israel military aircrew is affected by bruxism, and could frequently need a treatment with occlusal splints^12^. In fact, a previous study on military pilots affected by bruxism showed that this pathology affects their masticatory muscles determining a temperature reduction related with muscular hyperactivity and that acting on military pilots’ dental occlusion applying an occlusal splint, could help in achieving a relaxation of their facial muscular system observable by an increase of the masticatory muscles temperature^1^.

Thus, it is generally accepted that chronic stressful situations affect the stomatognathic apparatus altering the occlusal and muscular condition^13,14^ and a clear example could be represented by the peculiar stomatognathic condition observed in military pilots^1,5^.

In fact, should be important to assess if military pilots could present job-related adaptations of the stomatognathic apparatus performing an accurate analysis of dental occlusion of these subjects. T-scan system (T-Scan III, Tekscan Inc. Boston, MA, USA with T-Scan HD sensor) is one of the most precise^15,16^ and commonly used computerized analyzing systems to objectively assess dental occlusion status and the masticatory activity in a dynamic perspective^17–21^. The system records relative force values and objectively quantifies dynamic occlusal relationships by displaying numerical values for occlusion and disclusion times^20^. In particular, the Occlusion Time (OT) is a dynamic parameter defined as the elapsed time from initial tooth contact until maximum intercuspation is reached in centric occlusion. It is directly related with each subject’s occlusal contact pattern and occlusal stability^17^ and some studies have considered this dynamic parameter as capable of describing a subjects’ stable occlusion^22,23^. For example, a previous study of Baldini *et al*.^24^ demonstrated that subjects affected by temporomandibular disorders (TMD) have longer OT compared with healthy subjects suggesting that the presence of TMD is associated with a lower occlusal stability, while another study by Gumus *et al*.^25^ investigated the effect of an occlusal splint treatment in subjects affected by bruxism on the occlusion time and other T-Scan parameters and observed the absence of influence of bruxism on the occlusion time.

Today literature lacks information about the job-related adaptations of the stomatognathic apparatus in military pilots and thus, investigations are encouraged. The analysis of the occlusion time can be considered a meaningful parameter for evaluating their occlusal stability and dynamic relationships.

Furthermore, from a clinical point of view, considering that previous studies demonstrated that occlusal alterations in military pilots could influence their body posture^2^, analyzing these data could help the stomatologist in having a minimally invasive impact on the dental occlusion of military pilots with eventual conservative or prosthesis therapies.

Thus, the aim of this case-control study is to analyze the OT in a sample of high-performance military pilots affected by bruxism, compared with healthy non-pilot subjects.

## Methods

### Subjects

Fourteen military pilots of high-performance aircraft with 1000–3000 flight hours (all members of the P.A.N., the National Aerobatic Patrol of the Italian Air Force), all males (mean age 35.14 ± 2.32 years), and affected by bruxism, were enrolled in the study, and compared with an age matched control group of 14 male non-pilot subjects without signs or symptoms of bruxism (mean age 32.29 ± 4.84 years).

All the subjects were screened at the Institute of Aerospace Medicine «A. Mosso» in Milan (Italy). The ethical committee of the Institute approved the protocol that is in agreement with the declaration of Helsinki and all the enrolled subjects signed a consent form prior to their participation.

The screening included an anamnesis, oral examination, and Temporomandibular Joint (TMJ) evaluation of the subjects, to seek out wrong dental occlusion relationships or TMD. The screening confirmed that all the selected pilots were affected by bruxism.

In particular, the diagnosis of bruxism was attributed with patients self-reports and clinical inspections considering the following points: patient previous self-report of teeth grinding and consequent assessment of multiple grinding episodes during a period of two weeks, bed partner or parents report of teeth grinding episodes, presence of masticatory muscles hypertrophy, presence of indentation of the tongue or lip, presence of a *linea alba* on the inner cheek, damage of the dental hard tissues with mechanical wear of the teeth.

Both pilots and controls had to meet the following inclusion criteria: good general health according to their medical history, i.e. all the enrolled subjects had to be free from systemic or local diseases.

Other inclusion criteria were: absence of periodontal disease, absence of cast restorations and extensive occlusal restoration (less than 3 teeth with onlay restorations), occlusal Angle Class I or II, absence of TMD diagnosis, absence of previous orthodontic therapy, absence of crossbite, absence of previous facial or mandibular traumas, good compliance with oral hygiene, absence of neurologic disturbances, absence of orthognathic surgery.

Furthermore, the control subjects had to be free from bruxism.

### Procedure

The T-Scan III computerized occlusal analysis system (Tek-Scan Inc., Boston, MA, USA) was used to record the subjects’ OT during 4 mandibular opening-closing movements. The hardware is represented by a recording handle connected to the computer through a USB port. Held securely within the recording handle is a rigid fork-shaped plastic sensor holder, that should be placed in mouth in contact with the central maxillary incisors. The digital sensor has a thickness of 100 μm and is positioned flat between the dental arches during all the recording procedures. When a force is applied on the sensor, a voltage drop occurs on its conduction paths that is immediately recorded by the software and then displayed for each tooth contact.

Prior to any occlusal data acquisition, a proper sensitivity range was established according to the manufacturer’s recommendation, and the sensor conditioning procedures were performed for each participant.

During the procedure the patients sat upright in the dental chair with the Frankfurt plane parallel to the floor.

The OT were recorded by placing the sensor between the dental arches, and asking the patient to perform 4 mandibular opening-closing movements without clenching, in and out of complete intercuspation with a velocity of about 1 movement per second^17,24^. A single new sensor was used for each subject and all recordings were made by the same expert operator between 10:00 AM and 12:00 PM hours, in order to exclude any inter-rater and/or diurnal variability. Moreover, in order to compensate for individual variations in bite force, the T-scan sensor was recalibrated between patients prior to measurement according with the guidelines of the producer.

Thus, a mean OT value was calculated for each subject of both groups.

### Statistical analysis

An a priori sample power analysis based on a preliminary pilot study was conducted in order to achieve a minimum of 95% power with an alpha value of 0.05.

Two repeated recordings of mean OTs were obtained from 14 subjects to calculate the method error of the recordings using the Method Moment Estimator (MME)^26^.

Because of the reduced sample size, a non-parametric Mann Whitney U test was performed in order to analyze the differences between the mean OT of the two groups.

Results were considered to be significant at the 5% critical level (p < 0.05).

### Ethical approval

All procedures performed in this study involving human participants were in accordance with the ethical standards of the institutional and national research committee and with the 1964 Helsinki declaration and its later amendments or comparable ethical standards.

### Informed consent

Informed consent was obtained from all individual participants included in the study.

## Results

The actual post hoc calculated sample statistical power was higher than 99%. The method error calculated using the MME was equal to 0.017 s. Data are summarized as median and 25–75 percentiles in Table 1.

**Table 1:** Data summarized and statistical comparisons.

	AGE (years)		OCCLUSION TIME (s)	
	Mean	SD	Median	25–75%
Military Pilots	35.50	2.32	0.075	0.066–0.087
Control Group	32.29	4.84	0.429	0.038–0.057
p-value	n.s.		<0.0001	

Military pilots affected by bruxism showed a statistically significant lower (reduced of 85%) mean OT compared with healthy control subjects (p < 0.0001).

## Discussion

The accelerative stresses that daily affect military pilots determine a peculiar medical condition characterized by different work-related pathologies^7^. In particular, military pilots have an importantly increase incidence of bruxism, as confirmed by a previous study that found bruxism of clinical importance in the 71% of Israel military jet pilots and only in the 27% of the non-pilots aircrew members^11^. OT is directly related with patients’ occlusal contact pattern, and has been considered as a capable description of their occlusal stability and health^17,20,24^.

The present results showed a statistically significant, and clinically relevant, reduced OT in military pilots, compared with healthy non-pilot controls. In particular, the mean OT in military pilots was more than 6 times less than healthy subjects.

Although it could be hypothesized this finding to be related with the dental abrasion, typical of subjects affected by bruxism, it seems particularly interesting to compare this result with the findings of Gumus *et al*.^25^ that, as a secondary outcome of their study, similarly compared the OT of subjects affected by bruxism with a healthy group, without finding any statistically significant difference. Gumus *et al*.^25^ report an OT in bruxists of 0.144 (0.115–0.318), and in healthy not bruxist subjects of 0.220 (0.175–0.290), without significant differences. Thus, their results clarify that bruxism in not a mean confounding variable in the present study, as it does not affect the OT. This concept contributes to strengthen the conclusion of the present investigation, that the observed differences between the test and the control groups should be related to the employment of the subjects involved (i.e. as a professional pilot) rather than to the abrasion of the teeth and/or bruxism. In addition, it must be noted that the data from the two studies are quite different.

In the present study, the pilots showed a halved mean OT, compared to the bruxist group from Gumus *et al*.^25^ (respectively 0.076 s and 0.144 s) but the healthy controls from the present investigation show higher OT values than Gumus *et al*.^25^ (respectively 0.481 s and 0.220 s).

The reasons of such differences can not be clarified from the present investigation, as the subjects were all young adults (32.3 years in the present study *versus* 26.7 years in the study by Gumus *et al*.^25^) and the gender distribution should not affect the data^24^. In addition, regarding healthy non-pilot subjects, literature^17^ reports values even higher of about 0.69 s, suggesting the need of further investigations also in this field.

Considering that a difference in the procedure should affect both the samples data of a single study in the same manner, making their general findings comparable, it could be hypothesized that the differences between the two studies could be related with the particular category of military pilots and its peculiar physical condition.

In fact, it is plausible to assume that some differences could even exist among pilots, according to the type of training practiced, the number of flight hours and the type of aircraft. These differences could play a role in the stress level reflecting on their stomatognathic function. For this reason, the sample involved in the present investigation consists of particularly experienced and trained air force aerobatic pilots, all members of the same patrol, who presumably have the same experience and training program and a higher level of physical and psychological stress than other colleagues. Unfortunately, the present data do not allow to clarify this aspect, as no comparison was made with other groups of military pilots.

Furthermore, a limitation of the study could be the limited sample of the study that even if it could be considered with satisfying statistical power, could limit the generalizability of the results to the whole population of military pilots.

Thus, further studies are encouraged to enhance the scientific knowledge about the relationship between OT and bruxism and about the physical condition of each category of military pilot to protect their stomatognathic apparatus from job-related stresses and accelerations.

## Conclusions

Military pilots are a particular category of subjects typically affected by bruxism and are characterized by a highly reduced occlusion time compared to healthy non-pilot subjects.

This feature should not be related with the presence of bruxism but with their particular job-related physical condition because a previous study seem to suggest that there is no correlation between the presence of bruxism and a reduced occlusion time.

The dental implications of the peculiar clinical condition of military pilots as the influence of the category of military pilot on the results observed, should be further investigated.

## Data Availability

Data are stored by the corresponding author for privacy reasons but are available upon reasonable request.
